# Reduced health-related quality of life, fatigue, anxiety and depression affect COVID-19 patients in the long-term after chronic critical illness

**DOI:** 10.1038/s41598-024-52908-5

**Published:** 2024-02-06

**Authors:** Marion Egger, Corinna Wimmer, Sunita Stummer, Judith Reitelbach, Jeannine Bergmann, Friedemann Müller, Klaus Jahn

**Affiliations:** 1 Research Group, Department of Neurology, Schoen Clinic Bad Aibling, Kolbermoorer Strasse 72, 83043 Bad Aibling, Germany; 2 Institute for Medical Information Processing, Biometry and Epidemiology (IBE), Faculty of Medicine, LMU Munich, Pettenkofer School of Public Health, Munich, Germany; 3 German Center for Vertigo and Balance Disorders, University Hospital Grosshadern, Ludwig-Maximilians-Universität München, Munich, Germany

**Keywords:** Epidemiology, Quality of life, Prognosis, Outcomes research, Health care, Epidemiology

## Abstract

The term chronic critical illness describes patients suffering from persistent organ dysfunction and prolonged mechanical ventilation. In severe cases, COVID-19 led to chronic critical illness. As this population was hardly investigated, we evaluated the health-related quality of life, physical, and mental health of chronically critically ill COVID-19 patients. In this prospective cohort study, measurements were conducted on admission to and at discharge from inpatient neurorehabilitation and 3, 6, and 12 months after discharge. We included 97 patients (61 ± 12 years, 31% women) with chronic critical illness; all patients required mechanical ventilation. The median duration of ICU-treatment was 52 (interquartile range 36–71) days, the median duration of mechanical ventilation was 39 (22–55) days. Prevalences of fatigue, anxiety, and depression increased over time, especially between discharge and 3 months post-discharge and remained high until 12 months post-discharge. Accordingly, health-related quality of life was limited without noteworthy improvement (EQ-5D–5L: 0.63 ± 0.33). Overall, the burden of symptoms was high, even one year after discharge (fatigue 55%, anxiety 42%, depression 40%, problems with usual activities 77%, pain/discomfort 84%). Therefore, patients with chronic critical illness should receive attention regarding treatment after discharge with a special focus on mental well-being.

Trial registration: German Clinical Trials Register, DRKS00025606. Registered 21 June 2021—Retrospectively registered, https://drks.de/search/de/trial/DRKS00025606.

## Introduction

Advances in intensive care have substantially improved survival rates of critically ill patients^1^. However, these advances have also led to a growing population of patients suffering from persistent organ dysfunction and prolonged dependence on mechanical ventilation, a condition termed chronic critical illness (CCI)^2^. The underlying pathophysiology of CCI was suggested to be based on persistent inflammation, immunosuppression, and protein catabolism^3,4^. CCI can develop in all patients requiring treatment for acute medical, surgical, neurologic, or cardiac critical illness. It occurs especially often in older patients with sepsis, mechanical ventilation and underlying comorbid conditions^2^. As a consequence, CCI contributes to long-term mortality, extraordinary health-care costs, reduced long-term physical, psychological and cognitive functions, and diminished health-related quality of life^5–10^. The encompassing long-term disability of ICU survivors with impairments in physical function, psychological health, and cognition was previously described as post-intensive care syndrome (PICS)^11^.

The COVID-19 pandemic caused millions of infections worldwide. The disease caused by the severe acute respiratory syndrome coronavirus -2 (SARS-CoV-2) typically manifests as pneumonia and can lead to critical symptoms requiring treatment on ICU and mechanical ventilation^12–15^. Reconvalescence of ICU survivors after COVID-19 disease was shown to be protracted and large numbers suffered from health limitations even months after the infection. Within the first three months after discharge from ICU, around 90% still suffered from health state limitations^16–18^. Symptoms may even be prominent 1 year after ICU treatment, with physical, mental and cognitive impairments reported in 74%, 26% and 16% of the patients respectively^19^. In accordance with the reduced health state, health-related quality of life was also shown to be reduced in COVID-19 ICU survivors^14,20, 21^. Additionally, ICU admission and (duration of) mechanical ventilation were found to be predictors for a low health-related quality of life^21–23^.

Until now, only few studies investigated CCI in COVID-19 populations. Interestingly, reported mortality rates in COVID-19 CCI populations (90-day mortality = 28%^24^; 1-year mortality = 6.6%^25^) were substantially lower than previously described mortality rates in general CCI populations (1-year mortality = 44%^6^–54%^5^). Although mortality and survival are highly relevant, long-term outcome, prospect of life and quality of life became more and more important in intensive care medicine^26^. As treatment of CCI patients is highly resource-intensive, specific data on long-term trajectories are required for decision-making processes regarding resource allocation, critical care capacity and therapeutic options. Therefore, the outcome of COVID-19 patients with CCI is of high relevance, especially, as up to 50% of the investigated COVID-19 patients (being treated on ICU) were chronically critically ill^24,25^. Additionally, as health limitations are common in COVID-19 ICU survivors even after short durations of ICU therapy^14,16^, substantial and enduring health deficits can be expected in COVID-19 CCI patients^6,24^. However, up to now, there are no studies about the long-term outcome beyond the scope of pure survival.

Therefore, the objective of this study was to determine the physical and mental health and the health-related quality of life of chronically critically ill COVID-19 patients 3, 6 and 12 months after discharge from hospital.

## Methods

### Study population and setting

For this observational prospective cohort study, patients were recruited at the Schoen Clinic Bad Aibling, a center for inpatient neurorehabilitation in Germany with a focus on critically affected patients (ICU, early neurorehabilitation). Adult patients (≥ 18 years) with laboratory-confirmed COVID-19 (evaluated by real-time reverse transcriptase PCR) were eligible after the infectious stage and after being admitted to neurorehabilitation. Exclusion criteria were (1) insufficient (German) communication skills to complete the questionnaires and (2) patients receiving palliative care. For the analysis presented in this manuscript, only chronically critically ill COVID-19 patients were included. An American consensus-derived definition was applied to determine CCI^27^, whereby a minor adaption according to^9^ was used. This definition consists of at least 8 days in an ICU and one of six eligible clinical conditions (prolonged acute mechanical ventilation (≥ 96 h), tracheotomy, sepsis, severe wounds, stroke (including ischemic stroke and intracerebral hemorrhage), and traumatic brain injury). For the diagnosis of these conditions, the criteria of^9^ were applied.

Patients received approximately 100 min of multi-disciplinary neurorehabilitation therapies per day, including physio-, occupational-, dysphagia-, and breathing therapies, as well as neuropsychology. Duration of neurologic rehabilitation was distinct for every patient.

Part of this study population were described previously in a study on the clinical course during neurorehabilitation^20^ and a study about severe post-COVID-19 condition^28^.

The study was approved by the medical ethics committee of the Ludwig Maximilian University Munich according to the Declaration of Helsinki (project No. 20-0478). Written informed consent was obtained from all participants (or their legal guardians). The study was registered at the German Clinical Trials Register (DRKS00025606).

### Study visits and outcomes

Patients were included after admission to neurological rehabilitation (after discharge from ICU and after weaning from mechanical ventilation). Each of the 5 study visits (at study inclusion, at discharge from neurorehabilitation, and 3, 6, and 12 months after discharge) comprised a comprehensive set of questionnaires, functional tests, and questions about personal living conditions. The visits at study onset (visit 1 = V1) and at discharge (visit 2 = V2) from inpatient rehabilitation took place in person, visits 3, 4, and 5 (V3–V5) after discharge were conducted via structured telephone interviews and questionnaires sent by post. The study visits were conducted by trained and experienced study staff.

ICU treatment characteristics, complications, and pre-existing diseases were extracted from the medical records. To describe pre-existing diseases, a comorbidity index based on the Elixhauser classification system was used^29^. In order to investigate critical illness polyneuropathy and –myopathy, sensory and motor nerve conduction studies and needle electromyography (if applicable) were conducted after study inclusion.

This analysis focuses on the following assessments:

- The Fatigue Severity Scale-7 (FSS-7) is used to evaluate fatigue. The seven-item version has better psychometric properties than the nine–item version^30^. Score: 1–7. The cut-off ≥ 4 was interpreted as indicative of fatigue^31^.
- The Hospital Anxiety and Depression Scale (HADS) is a valid and reliable tool to measure anxiety and depression and was repeatedly used in critically ill patients^32^. Score: 0–21 each for anxiety and depression. A score of > 7 in each category was interpreted as clinically significant^33^.
- The EuroQol-5 dimensions-5 level (EQ-5D-5L) was used to measure health-related quality of life^34^. The index value for the German population ranges from − 0.205 (0 = health state equivalent to death; negative values = health state worse than death) to 1.000 (best health state)^35^. Patients who died after V1 were assigned a score of 0 in all further study visits. Additionally, the visual analogue scale (included in the EQ-5D-5L; 0–100) was used. 100 indicates the best imaginable state of health.
- The generic World Health Organization Disability Assessment Schedule 2.0 (WHODAS-12) measures health and disability and comprises the categories cognition, mobility, self-care, getting along, life activities, and participation. It is reliable, widely used and has good internal consistency^36^. The total score was converted into a percentage ((sum/48)*100): no (0–4%); mild (5–24%); moderate (25–49%); severe (50–95%); and complete (96–100%) disability.
- Frailty (Clinical Frailty Scale, score 1–9; 9 = deathly ill^37^), overall disability (modified Rankin Scale, score 0–6; 6 = death^38^) and dyspnea (modified Medical Research Council dyspnea scale, score 0–4; 4 = severest dyspnea^39^. For frailty and overall disability, a preclinical value was recorded retrospectively at visit 1.

### Statistical analysis

Categorical variables are presented as absolute values and percentages, continuous variables as mean ± standard deviation or median (quartile 1–quartile 3).

For the comparison of symptoms between different study visits, Friedman-Test was used as data were either non-parametric or did not follow normal distribution (as checked visually by means of QQ-plots). Effect sizes were calculated based on the Wilcoxon signed-rank test as Z statistic divided by square root of the sample size (r = Z/√N) for the comparisons of different study visits (in all patients with available data pairs).

Correlation between fatigue, depression, anxiety and health-related quality of life (index value) was calculated by spearman´s rank correlation coefficient and interpreted according to^40^.

Linear mixed-effect models for repeated measures were used to investigate the impact of preclinical health states and ICU treatment characteristics over time. Models were calculated separately for each of the outcomes, i.e. health related quality of life, fatigue, anxiety and depression. Variable selection was done based on literature research and expert knowledge. Multicollinearity was assessed for the independent variables using generalized variance inflation factors. The final model included age (included either annually or in decades for enhanced interpretability), sex, duration of mechanical ventilation (included either in days or with z-standardized values for enhanced interpretability), preclinical frailty, comorbidities (Elixhauser Comorbidity Index), obesity, diabetes, and ECMO treatment as fixed effects and a random intercept. A random intercept can be interpreted as individual variations of the referring outcome at baseline. Assumptions (normality of residuals, linearity and homogeneity of residual variance) were inspected visually for systematic violations. The adjusted intraclass-correlation coefficient (ICC) (the proportion of explained variance that can be attributed to the random effects) and the conditional R^2^ (the proportion of the explained variance of the full model, taking the fixed and random effects into account) were reported for each model. As a sensitivity analysis, models with additional random slopes were investigated. A random slope can be interpreted as individual variation in change of the outcome over time. Likelihood-ratio tests were used to compare models with random intercepts alone and models with additional random slopes. Although including a random slope significantly improved the model over a random intercept model, the conditional R^2^ and ICC were > 0.9 and therefore indicated a risk for overfitting. The results of the models with random slopes were included in Supplementary Table 2.

Statistical analyses were performed using R version 4.1.1 and IBM SPSS Statistics 19. The linear mixed-effect models was fitted using the ‘lme’ function of the ‘nlme’ package. The ICC and the R^2^ were calculated using the ‘icc’ and the ‘r2_nakagawa’ function of the ‘performance’ package. A *p*-value ≤ 0.05 was considered significant. Missing data was not replaced.

### Ethics approval and consent to participate

The study was approved by the medical ethics committee of the Ludwig Maximilian University Munich according to the Declaration of Helsinki (project No. 20-0478). Written informed consent was obtained from all participants (or their legal guardians).

## Results

**Figure 1:**
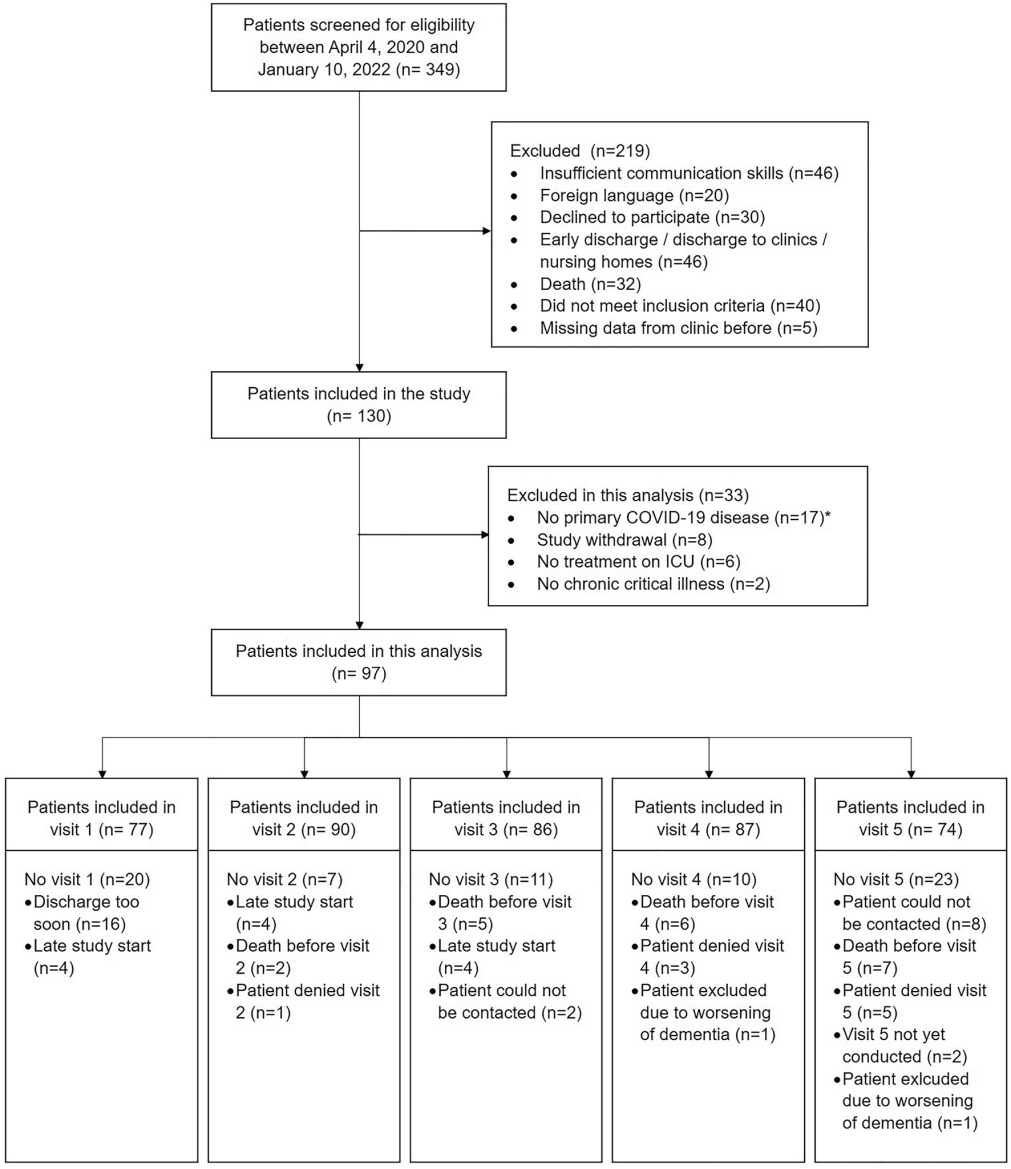
Flow chart. *no primary COVID-19 disease: patients hospitalized due to another neurologic disease who became infected with COVID-19 as a complication during the hospital stay.

A total of 349 patients were screened between April 2020 and January 2022. 130 were enrolled in the study from June 2020 until January 2022 and 97 were included in this analysis (Fig. 1). The median length of stay on ICU was 52 days (Table 1). Patients were admitted from 33 different ICUs and 52% patients were treated on two or three different ICUs. All patients required prolonged mechanical ventilation (median = 39 days), which led to the definition of CCI. The last telephone interview was conducted on February 1, 2023. Seven patients (7.2%) died over the course of the study. Patients frequently had suffered from sepsis (87.6%), acute respiratory distress syndrome (81.4%) and critical illness polyneuropathy and –myopathy (84.2%).

**Table 1:** Characteristics of included patients.

	Total n = 97
Age, years	61.1 ± 12.1, min/max: 29/90
Sex, women	30 (30.9%)
Length of hospitalization, days	110 (77.5–142.5); 117.0 ± 50.2
Length of ICU stay, days	52 (36–71); 55.7 ± 26.5
Length of mechanical ventilation, days	39 (22–54.5); 42.5 ± 24.3
Length of neurological rehabilitation, days	46 (28–68); 52.1 ± 32.9
Chronic critical illness—conditions	
Prolonged acute mechanical ventilation (≥ 96 h)	97 (100.0%)
Tracheotomy	73 (75.3%)
Sepsis	85 (87.6%)
Severe wounds	28 (28.8%)
Stroke	9 (9.3%)
Traumatic brain injury	0 (0.0%)
Complications	
Acute Respiratory Distress Syndrome (ARDS)	79 (81.4%)
Bacterial superinfection	62 (63.9%)
Dysphagia	50 (51.5%)
Acute kidney injury	30 (36.6%)
Delirium	31 (32.0%)
ECMO treatment	23 (23.7%)
Severe encephalopathy	15 (15.5%)
Guillain-Barré-Syndrome	2 (2.1%)
Critical illness polyneuropathy/myopathy	
Electrophysiological measurement conducted	76 (78.4%) (missing n = 21)
Time between infection and measurement, days	89.9 ± 36.4
Critical illness polyneuropathy/myopathy diagnosed	64 (84.2%)
Comorbidities	
Diabetes (all type II)	22 (22.7%)
Obesity	24 (24.7%)
Hypertension	47 (48.5%)
Elixhauser Comorbidity Index	3.1 ± 6.5, min/max: − 7/27
Preclinical status	
Frailty (Clinical Frailty Scale)	2 (1–3)
Disability (modified Rankin Scale)	0 (0–0)
Vaccination status at first SARS-CoV-2 infection	
No COVID-19 vaccination	83 (85.6%)
First COVID-19 vaccination	1 (1.0%)
Two/three COVID-19 vaccinations	6 (6.2%)
Missing	7 (7.2%)
Occupation	
Employed	40 (41.3%)
Self-employed	15 (15.5%)
Retired	34 (35.1%)
Volunteer work	4 (4.1%)
Housewife	2 (2.1%)
Unemployed	2 (2.1%)
Living conditions	
At home alone	20 (20.6%)
At home not alone (e.g., with family)	76 (78.4%)
Sheltered housing	1 (1.0%)
Relationship	
Married/in a relationship	72 (74.2%)
Single/divorced	16 (16.5%)
Widowed	9 (9.3%)
Cigarette smoking (missing n = 3)	
Current smoker	7 (7.5%)
Former smoker (quit a maximum of 10 years ago)	14 (14.9%)
Alcohol consumption (missing n = 2)	
Never	30 (30.9%)
Once per month	10 (10.3%)
2–4 times per month	20 (20.6%)
2–3 times per week	14 (14.4%)
4 times per week or more	21 (21.6%)
Discharge destination (n = 95; 2 deaths during rehabilitation)	
Further rehabilitation	21 (22.1%)
Home	64 (67.4%)
Home with (mobile) nursing service	5 (5.3%)
Other hospital	3 (3.2%)
Sheltered housing	1 (1.0%)
Nursing home	1 (1.0%)
Days from first positive PCR until…	
V1	86.2 ± 29.9
V2	119.2 ± 47.2
V3	226.2 ± 50.7
V4	309.6 ± 46.8
V5	504.5 ± 57.1
Days between ICU discharge and V1	15 (9–30); 22.8 ± 21.6
Living conditions at V3-V5 (n = 92; 5 deaths before V3)	
At home alone	18 (19.6%)
At home not alone (e.g., with family)	72 (78.2%)
Sheltered housing	1 (1.1%)
Nursing home	1 (1.1%)

Data are n (%), mean ± SD or median (quartile 1–quartile 3).

*ICU* intensive care unit, *ECMO* extracorporeal membrane oxygenation, *V1* Study onset after admission to neurorehabilitation, *V2* discharge from neurorehabilitation, *V3 / V4 / V5* 3 / 6 / 12 months after discharge from neurorehabilitation.

Table 2 shows the results of the outcomes from visit 1–5 (see Supplementary Table 1 for numbers of available data for every assessment and every time point). The general health state improved over time regarding the overall disability, frailty and dyspnea, as illustrated by the large effect sizes from visit 1–2 (which indicates the positive effect of the neurological rehabilitation) and visit 1–5. However, values for fatigue, anxiety and depression at visits 3–5 were higher than at visit 1 (shortly after discharge from ICU) and their prevalence increased since that time. Health-related quality of life improved since study onset, although the maximum value was found to be at discharge from rehabilitation (index value 0.73 ± 0.20).

**Table 2:** Results of the questionnaires over the course of the study.

	Visit 1 at study onset	Visit 2 at discharge	Visit 3: 3 months after discharge	Visit 4: 6 months after discharge	Visit 5: 12 months after discharge	Friedman-Test V1–V5	Effect size			
							V1–V2	V2–V3	V1–V5	V3–V5
Modified Rankin Scale	4 (3–4)	2 (2–3)	3 (2–3)	2 (1–3)	2 (1–3)	χ^2^ (4) = 64.06, *p* < .001	0.76	0.07	0.63	0.39
Clinical Frailty Scale	6 (6–7)	5 (4–6)	5 (3–6)	4 (3–5)	4 (3–5)	χ^2^ (4) = 115.8, *p* = < .001	0.85	0.26	0.85	0.57
Modified Medical Research Council Dyspnea Scale	3 (2–4)	2 (1–2)	1 (0–2)	1 (0–2)	1 (0–2)	χ^2^ (4) = 32.39, *p* < .001	0.84	0.44	0.80	0.12
FSS-7	2.7 ± 1.5	2.8 ± 1.7	3.8 ± 2.0	3.9 ± 2.0	4.0 ± 1.9	χ^2^ (4) = 29.38, *p* < .001	0.07	0.47	0.52	0.01
Fatigue ≥ 4	18 (23.7%)	20 (22.5%)	34 (44.7%)	38 (53.5%)	35 (54.7%)					
HADS										
Anxiety	5 (2–8)	4 (1–6)	7 (3–10)	6 (3–9)	7 (3–11)	χ^2^ (4) = 10.95, p = .027	0.33	0.54	0.14	0.02
Anxiety > 7	23 (29.9%)	19 (21.6%)	32 (42.7%)	26 (37.1%)	27 (42.2%)					
Depression	4 (2–8)	3 (1–7)	6 (3–10)	5 (2–9)	6 (3–9)	χ^2^ (4) = 12.32, *p* = .015	0.29	0.53	0.26	0.03
Depression > 7	20 (26.0%)	14 (15.9%)	32 (42.7%)	20 (28.6%)	25 (39.1%)					
EQ-5D-5L										
Visual analogue scale	51.2 ± 20.2	64.1 ± 18.8	56.0 ± 21.2	60.9 ± 22.1	59.0 ± 23.9	χ^2^ (4) = 13.70, *p* = .008	0. 55	0. 37	0. 26	0. 24
Index value	0.53 ± 0.29	0.73 ± 0.20	0.63 ± 0.29	0.64 ± 0.30	0.63 ± 0.33	χ^2^ (4) = 10.76, *p* = .029	0.63	0.32	0.26	0.06
Problems with walking around	74 (96.1%)	65 (74.7%)	58 (75.3%)	46 (63.9%)	42 (67.7%)					
Problems with washing/dressing	65 (84.4%)	43 (49.4%)	38 (49.4%)	37 (51.4%)	25 (40.3%)					
Problems with usual activity	64 (83.1%)	45 (51.7%)	62 (80.5%)	49 (68.1%)	48 (77.4%)					
Pain or discomfort	62 (80.5%)	71 (81.6%)	64 (83.1%)	64 (88.9%)	52 (83.9%)					
Anxiety or depression	43 (55.8%)	23 (26.4%)	41 (53.2%)	38 (52.8%)	34 (54.8%)					
WHODAS-12 score, %	N/A	N/A	38.1 ± 24.3	33.7 ± 25.0	34.6 ± 23.7	χ^2^ (2) = 4.41, *p* = .110	N/A	N/A	N/A	0.20

Data are n (%), mean ± SD or median (quartile 1–quartile 3); FSS-7 = Fatigue-Severity-Scale-7; HADS = Hospital Anxiety and Depression Scale; EQ-5D-5 l = EuroQol-5 dimensions-5 level; WHODAS-12 = World Health Organization Disability Assessment Schedule 2.0–12 items; The effect size was calculated with $r=z/\sqrt{N}$. Effect sizes are small (≥ 0.1), moderate (≥ 0.3) or large (≥ 0.5) according to Jacob Cohen: Statistical Power Analysis for the Behavioral Sciences (1988), pp. 79–81.  unchanged  Improvement View Deterioration; Due to missing values, sample size included in the Friedman-test for V1–V5 differs per assessment: modified Rankin Scale n = 56; Clinical Frailty Scale n = 50; Modified Medical Research Council Dyspnea Scale n = 18; FSS-7 n = 38; HADS n = 38; EQ-5D-5L Index n = 43; EQ-5D-5L Visual Analogue Scale n = 37; WHODAS-12 n = 49.

**Figure 2:**
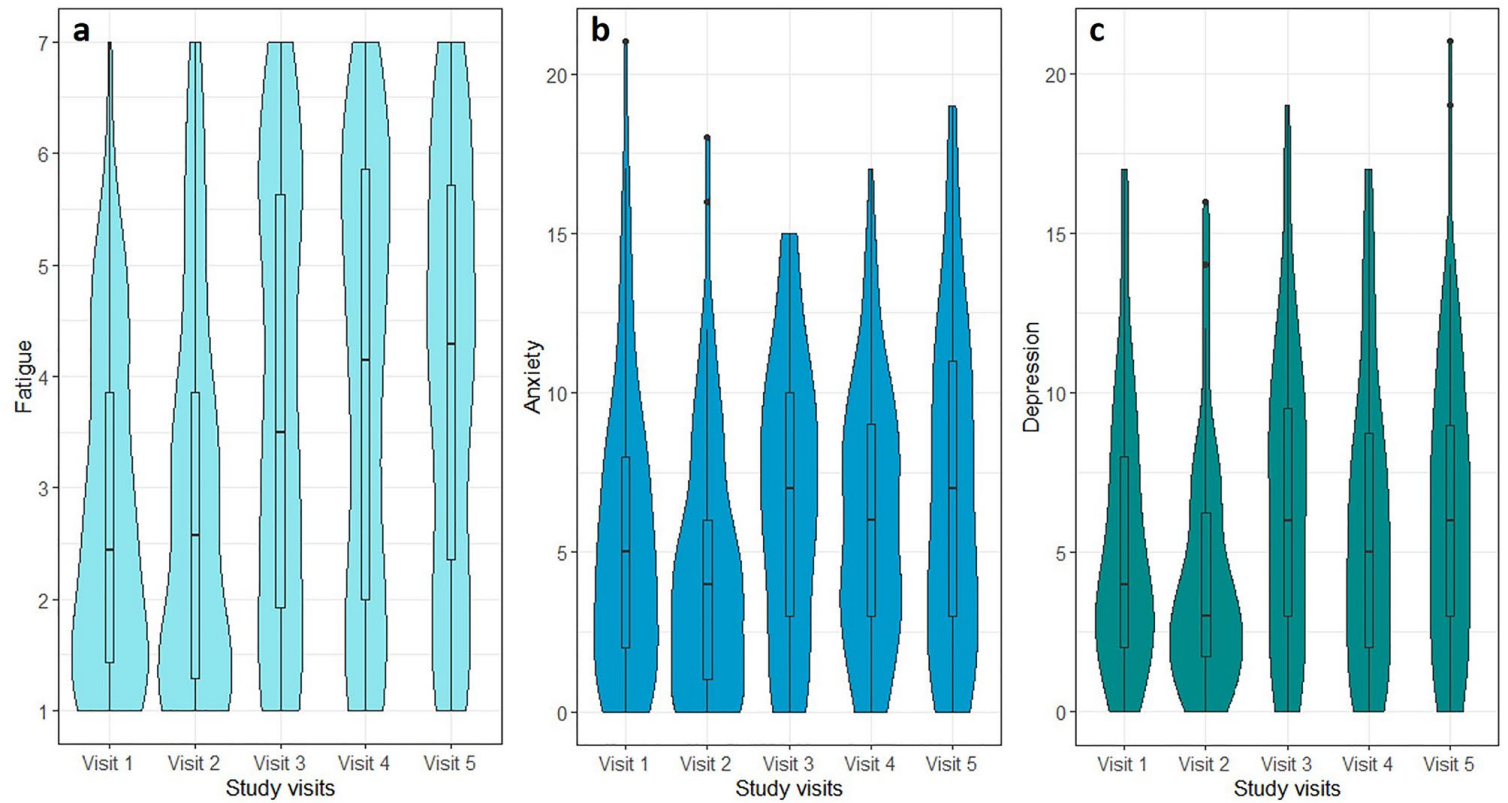
Violin plots including boxplots for comparing probability distributions of fatigue (**a**), anxiety (**b**) and depression (**c**) over the time course of visits 1–5. The remarkable change of data between visits 2 and 3 (i.e. the distribution) is clearly visualized by the plots’ change of shape and the increased medians and interquartile ranges within the boxplots.

**Figure 3:**
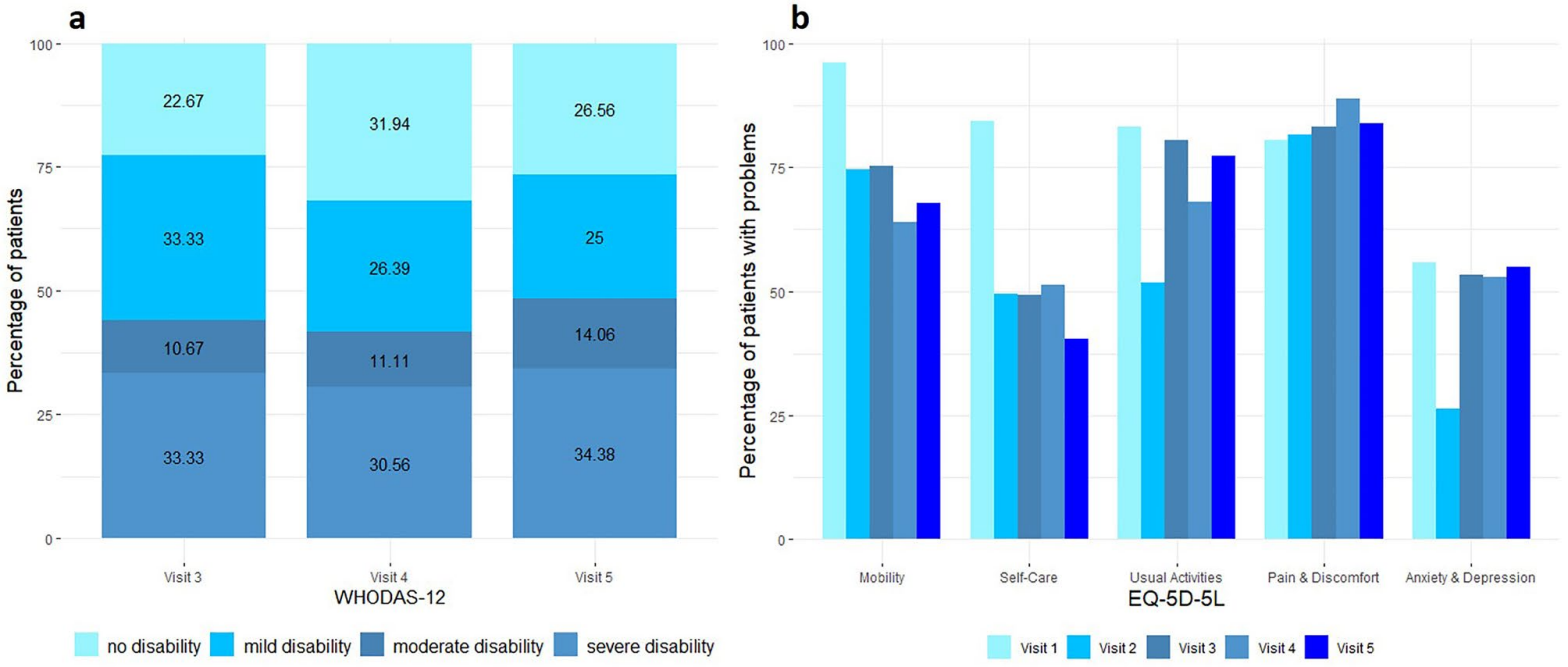
Percentage of patients with different degrees of disability according to WHODAS-12 (**a**) and with problems according to the five domains of the EQ-5D–5L (**b**) over the time course of visits 1–5.

The largest changes were found to be between visit 2 (at discharge) and visit 3 (3 months after discharge), especially regarding the prevalence of fatigue, anxiety, and depression. This is also illustrated in Fig. 2, in which the shape of the violin plots clearly changes from visit 2 to visit 3. Corresponding to this symptom deterioration, health-related quality of life decreased significantly from visit 2 to visit 3. Especially the frequency of problems regarding usual activities, anxiety and depression increased within this timeframe (Fig. 3). Overall, this symptom deterioration is also displayed by moderate to large effect sizes (r = 0.32–0.53).

Between visit 3–5, the burden of symptoms stayed mostly unchanged (small effect sizes for most outcomes, except for the Clinical Frailty Scale and modified Rankin Scale). Three months after discharge, the majority of patients suffered from pain or discomfort (83.1%) and faced most problems regarding usual activities (80.5%) and walking around (75.3%). The frequency of problems remained high until visit 5 (Fig. 3). Approximately one third suffered from severe disability, whereas approximately 50% had no or only mild disability according to WHODAS-12 (Fig. 3). Although severity of dyspnea decreased over time, nearly two thirds still had problems with breathing (62.9%, data not shown) at visit 5.

Correlations of fatigue with depression (r_s_ = 0.55, *p* < 0.001), anxiety (r_s_ = 0.60, *p* < 0.001) and health-related quality of life (r_s_ = − 0.33, *p* < 0.001) were fair to moderate.

Results of the linear mixed-effect models are displayed in Table 3. In the final model, time since disease onset (β = 0.09–0.18, *p* < 0.01), mechanical ventilation (β = − 0.06, *p* = 0.01 (z-standardized)) and preclinical frailty (β =  − 0.07, *p* = 0.02) were significant predictors for health-related quality of life. Time was also a significant predictor for the outcomes fatigue (β = 1.02–1.18, *p* < 0.0001), anxiety (β = − 1.12–1.20, *p* < 0.04) and depression (β = 1.09–1.50, *p* < 0.04). All model confirmed the significant increase of fatigue, anxiety and depression over time compared to the levels at visit 1. Additionally, a significant association of obesity with anxiety was found (β =  − 2.18, *p* = 0.02).

**Table 3:** Predictors for health-related quality of life, fatigue and mental health (linear mixed model).

	Health Index		Fatigue		Anxiety		Depression	
	Fix Eff	95% CI	Fix Eff	95% CI	Fix Eff	95% CI	Fix Eff	95% CI
Intercept	0.83****	0.55–1.11	3.45***	1.49–5.42	8.30***	3.53–13.08	4.44	− 0.38–9.27
Age								
Age [years]	− 0.00	− 0.00–0.00	− 0.01	− 0.04–0.02	− 0.06	− 0.13–0.01	− 0.01	− 0.09–0.06
Age [decades]	− 0.01	− 0.05–0.03	− 0.12	− 0.40–0.17	− 0.56	− 1.25–0.14	− 0.15	− 0.85–0.55
Sex = male	0.02	− 0.09–0.12	− 0.49	− 1.19–0.21	− 0.70	− 2.41–1.01	0.02	− 1.88–1.57
Mechanical ventilation								
Duration MV [days]	− 0.00*	− 0.00– (− 0.00)	− 0.00	− 0.01–0.01	0.01	− 0.02–0.04	0.01	− 0.02–0.04
Duration MV [z-standardized]	− 0.06*	− 0.10– (− 0.01)	− 0.02	− 0.35–0.31	0.24	− 0.55–1.04	0.25	− 0.55–1.05
Time visit 2 (vs. time visit 1)	0.18****	0.12–0.25	0.08	− 0.33–0.49	− 1.12*	− 2.01–(− 0.22)	− 0.90	− 1.81–0.02
Time visit 3 (vs. time visit 1)	0.09**	0.03–0.16	1.02****	0.59–1.45	1.20*	0.26–2.13	1.50**	0.54–2.46
Time visit 4 (vs. time visit 1)	0.09**	0.02–0.15	1.15****	0.70–1.59	0.54	− 0.43–1.51	0.72	− 0.28–1.71
Time visit 5 (vs. time visit 1)	0.10**	0.03–0.16	1.18****	0.73–1.63	1.05*	0.06–2.04	1.09*	0.07–2.10
Comorbidities	0.00	− 0.00–0.01	0.01	− 0.04–0.06	− 0.04	− 0.16–0.07	0.01	− 0.11–0.13
Obesity = yes	0.05	− 0.06–0.16	− 0.28	− 1.03–0.47	− 2.18*	− 4.02–(− 0.33)	− 1.43	− 3.30–0.43
Diabetes = yes	− 0.06	− 0.17–0.05	0.10	− 0.66–0.86	0.25	− 1.60–2.11	0.25	− 1.62–2.13
Preclinical frailty	− 0.07*	− 0.12–(− 0.01)	0.13	− 0.23–0.49	0.50	− 0.39–1.38	0.64	− 0.25–1.53
ECMO = yes	− 0.10	− 0.21–0.01	0.64	− 0.16–1.44	1.04	− 0.91–2.99	1.25	− 0.73–3.22
Adjusted ICC	0.445		0.479		0.551		0.542	
Conditional R^2^	0.541		0.544		0.600		0.582	

Fix Eff = fixed effects; 95% CI = 95% Confidence interval; Duration MV = Duration of mechanical ventilation in days; Comorbidities were measured by the Elixhauser Comorbidity Index; *ECMO* extracorporeal membrane oxygenation; *ICC* intraclass-correlation coefficient; *p < .05. **p < .01. ***p < .001. ****p < .0001. For enhanced interpretability, fixed effects were additionally calculated for models with age per decade and z-standardized values for mechanical ventilation instead of age (annually) and mechanical ventilation in days.

Significant values are in bold.

## Discussion

We investigated the physical and mental health, and the health-related quality of life of chronically critically ill COVID-19 patients 3, 6 and 12 months after discharge from neurorehabilitation. We showed that the overall disability, frailty and dyspnea improved after admission to neurologic rehabilitation. However, the prevalence of fatigue, anxiety, and depression increased over time, especially between discharge and the first study visit three months later, and remained on a high level until the last study visit one year after discharge. Accordingly, health-related quality of life was shown to be limited without noteworthy improvement until the last study visit. Time (since disease onset) had a significant influence on the outcomes anxiety, depression, fatigue and health-related quality of life.

### Post-intensive care syndrome (PICS) and symptom prevalences

Patients after critical illness in general frequently suffer from physical, mental and cognitive symptoms in the long-term, which is described as PICS^7^. As an example, PICS problems were reported to be present in 56% of patients with respiratory failure or shock 12 months after hospital discharge^41^. PICS was also frequently described in patients after severe COVID-19 disease^42^ and percentages were similar, as PICS was reported in 61% of patients 13.5 months after ICU discharge^43^. As we did not plan to investigate PICS in our study, no cognitive evaluation was included in the outcome parameters. However, physical function and mental health were investigated by the EQ-5D-5L and the HADS, which are both recommended assessments to detect PICS^44^. 12 months after discharge from rehabilitation, anxiety and depression were present in 42% and 39% of patients, respectively. 68% reported problems with walking and even 84% reported pain or discomfort, wherefore we can conclude that the majority of our participants suffered from PICS, even more than one year after the infection.

Previously described long-term outcomes of COVID-19 ICU survivors included a variety of symptoms, which are summarized by the terms post COVID-19 condition (according to the WHO’s case definition) or post-COVID-19 syndrome (according to the NICE guideline on long COVID) and often meet the diagnostic criteria for PICS. However, the symptom load of the post COVID-19 condition is substantially higher in our CCI cohort (Supplementary Table 3). Heesakkers et al.^19^ reported anxiety and depression in 18% of patients one year after ICU (median 18.5 days on ICU), compared to ~ 40% in our cohort. These authors also reported a median frailty of 2 (vs. 4 in our cohort). Hodgson et al.^14^ investigated the outcome of a cohort at 6 months (median 8.3 days on ICU). In this cohort, only 5% suffered from a severe disability according to the WHODAS-12, compared to 31% in our cohort. Accordingly, health-related quality of life was substantially lower in our cohort (visual analogue scale, 61 ± 22), compared to the Hodgson-cohort (median = 70 (IQR 60–85)). These examples illustrate the diverging convalescence of patients, which is most likely due to differences in the length of ICU treatment and mechanical ventilation (median 13–14 days^14,19^ compared to 39 days in our cohort).

PICS and an impaired health status were also frequently described in patients after critical illness in general (non-COVID-19 diseases)^7^. Just like in COVID-19 patients, symptom load seems to be associated with the length of stay on ICU or the duration of mechanical ventilation in patients with general critical illness (Supplementary Table 3). In patients after sepsis (median duration on ICU 10 days), 6 months after ICU admission, anxiety and depression were only reported in 26% and 21% (compared to our reports of 37% and 29%).^45^ Accordingly, 12 months after ICU, health-related quality of life (expressed by the visual analogue scale of the EQ-5D-5L) was 66 (44–80) in patients with a median of 8 days on ICU^46^ and 75 (60–89) in patients with a median of 2 days on ICU^47^. Both values were substantially higher compared to our cohort (59 ± 24). In patients with CCI, the quality of life is more similar to ours (Supplementary Table 3). Thomas et al. reported a value of 60 (IQR 29) in patients after 41 days on ICU^48^, Gardner et al. reported a value of 49 ± 7 in patients after 21 days on ICU^5^. According to our results in comparison with the literature it might be assumed that ICU parameters have a greater influence on CCI outcome than the disease leading to ICU admission. However, up to now, studies in patients with CCI examining patient-reported outcomes / PICS and their predictors are scarce.

### Health-related quality of life

Health-related quality of life improved after ICU discharge, but then remained rather unchanged at a low level. This level is substantially lower compared to an equally aged general population in Germany (index value 0.87 ± 0.20; our cohort at visit 5 0.63 ± 0.33). This lack of improvement is contrary to published studies, in which improvements in health-related quality of life from 3 to 12 months in critically ill COVID-19 patients were reported^23,49,50^. As the duration of mechanical ventilation was shown to negatively influence health-related quality of life^50^, this might be one explanation for the low level reported in our cohort with particularly long durations of mechanical ventilation.

The peak quality of life at discharge from inpatient care in our study might be explained by the patients’ improvements of independence in activities of daily living and happiness at finally going home. Additionally, at visit 2, the patients were still in the sheltered environment of the neurorehabilitation center with offers of help, prepared meals and accessible surroundings. The return to home with its responsibilities and being on one’s own might have been challenging and therefore might cause a reduction of health-related quality of life. The same might apply for fatigue. Inability to manage activities of daily living without help and being confronted with the prior healthy living conditions might further lead to anxiety and depression.

### Fatigue, anxiety and depression

Although COVID-19 ICU survivors usually improve their physical functions over time^49,51^, fatigue prevalence may rise, as observed in our cohort. Mazza et al. made the same observation in a cohort of hospitalized COVID-19 patients (~ 8% of ICU admissions), where fatigue increased from 22 to 34% from 1 to 12 months after COVID-19^52^. Likewise, the results of a meta-analysis (68 studies, hospitalized and non-hospitalized patients) indicated no significant improvement of fatigue frequency ≥ 6 months compared to < 6 months after COVID-19 infection^53^. Two studies with non-COVID-19 CCI patients also concluded that time after discharge had no influence on fatigue severity^54,55^. Regarding anxiety and depression after COVID-19, different trajectories were described^56^. Rosa et al. showed a slight increase of symptoms of anxiety (16–25%) and depression (15–20%) from 3 to 12 months^23^. Vlake et al. and Lorent et al. (median ICU stay 13/18 days) reported unimproved severities of anxiety and depression over the course from 1.5 to 6 months and 3–12 months after hospital discharge, respectively^51,57^. In contrast, Gramaglia et al. described a significant reduction of anxiety and depression symptoms from 4 to 12 months after discharge in their less affected cohort (~ 12% ICU admissions)^58^. In a review, several studies were mentioned in which disease severity was a risk factor for anxiety and depression^56^, which speaks for the high percentages in our CCI cohort.

### Inflammatory processes

Inflammatory parameters are subject of several COVID-19 investigations. One review reported elevations in at least one measure of inflammation in 13 of 14 studies. Additionally, in 9 of 14 studies, proinflammatory markers and persistent fatigue and/or cognitive dysfunction were present^53^. Furthermore, it was reported that chronic fatigue, anxiety and depression of COVID-19 patients share the same pathophysiological mechanisms, which have a strong association with increased oxidative toxicity, lowered antioxidant defenses and inflammatory signs^59^. In addition to COVID-19, persistent inflammation is also one underlying pathophysiology of CCI and PICS^3,4, 7^. Furthermore, systemic inflammation is the primary cause of critical illness polyneuropathy and myopathy^60^, which was diagnosed in 84% of our CCI patients. Therefore, inflammatory processes might be one explanation for the extraordinarily high percentages of fatigue, anxiety and depression in our cohort of CCI patients after COVID-19.

## Limitations

We are aware that our study may have several limitations. First, our study was monocentric and the sample size was rather small; additionally, some patients did not participate in every study visit, so the sample size per assessment and per study visit differs and is reduced. Thus, larger multicenter cohorts with CCI patients are needed to confirm our findings. Second, our patients were largely unvaccinated (as most got infected before vaccination got available). As vaccinations was shown to have protective effect on post-COVID-19 condition^61^, symptoms might differ in CCI COVID-19 populations with vaccination. Furthermore, symptoms of anxiety and depression were shown to be more frequent in patients with previous psychiatric history^56^, which was not assessed in our study. Additionally, we were not able to include CCI patients with non-COVID-19 diagnoses as a control group, thus we cannot conclude that the reported symptoms are specific for COVID-19.

## Conclusions

Persons with CCI associated with COVID-19 suffer greatly from fatigue, anxiety, depression and low health-related quality of life, even one year after discharge from hospital. Improvements were found regarding basic functional capacities and dyspnea. In contrast, fatigue, mental health and health-related quality of life deteriorated over time or remained unchanged at an undesirable level. This analysis showed a higher burden of symptoms compared to other studies with shorter durations of ICU treatment and mechanical ventilation. Therefore, patients with CCI in COVID-19 require adapted therapies and supportive structures even several months after discharge. Special attention should be paid to this group in research and medical treatment.

### Supplementary Information

Supplementary Information.

## Data Availability

The datasets used and/or analysed during the current study are available from the corresponding author on reasonable request.

## Abbreviations

CCI: Chronic critical illness
ICU: Intensive care uni﻿t
PICS: Post-intensive care syndrome
