# Supplementary material for: Reduced health-related quality of life, fatigue, anxiety and depression affect COVID-19 patients in the long-term after chronic critical illness
Source: Sci Rep. 2024 Feb 6;14:3016. doi: 10.1038/s41598-024-52908-5 (PMC10847136; doi:10.1038/s41598-024-52908-5)
Supplement: Supplementary file 1 — Supplementary Information. [file 41598_2024_52908_MOESM1_ESM.pdf]

Supplementary Information

Reduced Health-Related Quality of Life, Fatigue, Anxiety and Depression affect COVID-19 Patients in the Long-Term after Chronic Critical Illness

Marion Egger*, Corinna Wimmer, Sunita Stummer, Judith Reitelbach, Jeannine Bergmann, Friedemann Müller, Klaus Jahn

*Corresponding author:

Marion Egger, M.Sc.
Schoen Clinic Bad Aibling, Department of Neurology, Research Group
Kolbermoorer Strasse 72, 83043 Bad Aibling, Germany
Phone +49 8061 / 903-1954
E-Mail: megger@schoen-klinik.de

Supplementary Table 1 Available number of data for every assessment at every time point

	**Visit 1 at study onset**	**Visit 2 at discharge**	**Visit 3 3 months after discharge**	**Visit 4 6 months after discharge**	**Visit 5 12 months after discharge**	**Friedman-test V1-V5**
Modified Rankin Scale	77	92	88	91	74	56
Clinical Frailty Scale	77	90	83	84	67	50
Modified Medical Research Council Dyspnea Scale	33	54	61	63	64	18
FSS-7	76	89	76	71	64	38
HADS						
Anxiety	77	88	75	70	65	38
Depression	77	88	75	70	65	38
EQ-5D-5L						
Visual Analogue Scale	77	91	82	72	63	37
Index value	77	91	76	78	69	43
WHODAS-12 Score	N/A	N/A	76	72	66	49

Data are absolute numbers; FSS-7 = Fatigue-Severity-Scale-7; HADS = Hospital Anxiety and Depression Scale; EQ-5D-5l = EuroQol – 5 dimensions – 5 level; WHODAS-12 = World Health Organization Disability Assessment Schedule 2.0 – 12 items;

Supplementary Table 2 Predictors for health-related quality of life, fatigue and mental health (linear mixed model including random slopes)

	Health Index		Fatigue		Anxiety		Depression	
	Fix Eff	95% CI	Fix Eff	95% CI	Fix Eff	95% CI	Fix Eff	95% CI
Intercept	0.77****	0.52-1.02	3.05**	1.22-4.88	8.77***	4.22-13.32	4.11	-0.53-8.75
**Age**								
Age [years]	0.00	-0.00-0.00	-0.01	-0.04-0.01	-0.06	-0.13-0.00	-0.01	-0.08-0.06
Age [decades]	-0.00	-0.04-0.03	-0.14	-0.40-0.13	-0.65	-1.31-0.01	-0.11	-0.79-0.56
Sex = male	-0.01	-0.10-0.08	-0.19	-0.84-0.47	-0.37	-1.98-1.25	0.02	-1.63-1.68
**Mechanical ventilation**								
Duration MV [days]	-0.00**	-0.00-0.00	-0.00	-0.02-0.01	0.02	-0.02-0.05	0.01	-0.02-0.04
Duration MV [z-standardized]	-0.05**	-0.09-(-0.01)	-0.06	-0.36-0.25	0.39	-0.37-1.15	0.30	-0.47-1.06
Time visit 2	0.19****	0.13-0.24	0.11	-0.22-0.44	-1.11**	-1.88-(-0.33)	-0.97**	-1.7-(-0.24)
Time visit 3	0.09*	0.02-0.17	0.96***	0.47-1.45	1.13*	0.09-2.16	1.36*	0.26-2.47
Time visit 4	0.09*	0.01-0.16	1.14****	0.69-1.60	0.68	-0.37-1.72	0.73	-0.35-1.81
Time visit 5	0.10*	0.01-0.19	1.21****	0.74-1.67	1.08	-0.16-2.33	1.02	-0.19-2.24
Comorbidities	0.00	-0.00-0.01	0.01	-0.03-0.05	0.02	-0.09-0.13	0.00	-0.12-0.11
Obesity = yes	0.05	-0.04-0.15	-0.18	-0.89-0.52	-2.47**	-4.21-(-0.72)	-1.72	-3.50-0.06
Diabetes = yes	-0.05	-0.15-0.04	0.32	-0.38-1.02	-0.11	-1.87-1.64	0.11	-1.68-1.91
Preclinical frailty	-0.05*	-0.10-(-0.00)	0.26	-0.08-0.61	0.31	-0.53-1.14	0.68	-0.18-1.54
ECMO = yes	-0.06	-0.16-0.04	0.60	-0.14-1.34	0.65	-1.20-2.51	1.26	-0.62-3.15
Adjusted ICC	0.911		0.907		0.918			0.907
Conditional R^2	0.923		0.919		0.927			0.916

Fix Eff = fixed effects;95% CI = 95% Confidence interval; Duration MV = Duration of mechanical ventilation in days; ECMO = extracorporeal membrane oxygenation; ICC= Intraclass-correlation coefficient; *p < .05. **p < .01. ***p < .001. ****p < .0001. For enhanced interpretability, fixed effects were additionally calculated for models with age per decade and z-standardized values for mechanical ventilation instead of age (annually) and mechanical ventilation in days.

Supplementary Table 3 Symptoms after critical illness due to COVID-19 and non-COVID-19

Publication	Evaluation time	Disease	ICU length [days]	MC length [days]	EQ-5D-5L VAS	Anxiety [HADS]	Depression [HADS]	Problems with walking, activities of daily living, pain & discomfort	WHODAS-12 Score %
Egger et al. (current study as comparison)	3,6,12 months after discharge	COVID-19	52 (36-71)	39 (22-55)	3 months: 56.0 ± 21.2<br>6 months: 61±22<br>12 months: 59.0±23.9	6 months: 37%<br>12 months: 42%	6 months: 29%<br>12 month: 39%	6 months:<br>Problems with walking 64%<br>Problems with ADL 68%<br>Pain / discomfort 89%<br>12 months:<br>Problems with walking 68%<br>Problems with ADL 78%<br>Pain / discomfort 84%	6 months: 33.7±25.0
**COVID-19**									
Hodgson et al. 2021[1]	6 months after ICU admission	COVID-19	8.3 (3.6-19)	13 (5-19)	70 (60-85)	20%	20%	Problems with walking 42%<br>Problems with ADL 44%<br>Pain / discomfort 50%	10.4 (2.1–22.9)
Cavalleri et al. 2022[2]	12 months after ICU discharge	COVID-19	8.5 (4.5-20)	13 (8-23)	73 (60-80)				
Heesakkers et al. 2022[3]	12 months after ICU treatment	COVID-19	18.5 (11-32)	14 (8-22)		18%	18%		
**Non-COVID-19**									
Hodgson et al. 2022[4]	6 months after ICU admission	Sepsis	9.8 (5.7-14.9)	5.5 (2.7-9.6)	66.1±20.7	26%	21%	Problems with walking 47%<br>Problems with ADL 69%<br>Pain / discomfort 53%	26.1±22.1
Gardner et al. 2019[5]	3,6,12 months after sepsis onset	Sepsis	21 (15-39)	Not given	3 months; 52±4.5<br>6 months: 50±3.9<br>12 months: 49±6.8				

Griffiths et al. 2013[6]	6 and 12 months after ICU admission	Mainly Pneumonic , septic shock	8 (5-16)	4 (2-11)	6 months:<br>64 (46-80)<br>12 months:<br>66 (44-80)			6 months:<br>Problems with walking 59%<br>Problems with ADL 69%<br>Pain / discomfort 73%<br>12 months:<br>Problems with walking 55%<br>Problems with ADL 65%<br>Pain / discomfort 69%	
Cavalleri et al. 2022[2]	12 months after ICU discharge	Medical + surgical critical illlness	2 (2-4)	1 (1-2)	75 (60-89)				
Thomas et al. 2018[7]	6 and 12 months after start of neurologic rehabilitatio n	ICUAW defined by CIP/CIM	41 (IQR 30)	53 (IQR 42)	6 months:<br>60 (IQR 30)<br>12 months:<br>60 (29)				
Myhren et al. 2010[8]	12 months after ICU discharge	Medical, surgical, trauma	12.0 (95% CI 10.3-13.8)	11.0 (95% CI 9.3-12.7)		33%	27%		
Rattray et al. 2005[9]	6 and 12 months after hospital discharge	Gastro-intestinal, respiratory, trauma, vascular & others	5.9 (2.2-13.0)	Not given		6 months:<br>41%<br>12 months:<br>45%	6 months:<br>26%<br>12 months:<br>27%		
Hatch et al. 2018[10]	3 and 12 months after discharge from ICU	Level 3 care on ICU > 24h	3 (2-6)	Not given		46%	40%		

ICU= Intensive care unit; MC=mechanical ventilation; EQ-5D-5l = EuroQol – 5 dimensions – 5 level; IQR = Interquartilerange; CI= Confidence Interval

References for Supplementary Table 3

1 Hodgson, C. L. *et al.* The impact of COVID-19 critical illness on new disability, functional outcomes and return to work at 6 months: a prospective cohort study. *Critical Care* **25**, 382, doi:10.1186/s13054-021-03794-0 (2021).

2 Cavalleri, J. *et al.* One-Year Functional Decline in COVID-19 and Non-COVID-19 Critically Ill Survivors: A Prospective Study Incorporating a Pre-ICU Status Assessment. *Healthcare* **10**, 2023 (2022).

3 Heesakkers, H. *et al.* Clinical Outcomes Among Patients With 1-Year Survival Following Intensive Care Unit Treatment for COVID-19. *Jama* **327**, 559-565, doi:10.1001/jama.2022.0040 (2022).

4 Hodgson, C. L. *et al.* Comparison of 6-month outcomes of sepsis versus non-sepsis critically ill patients receiving mechanical ventilation. *Critical Care* **26**, 174, doi:10.1186/s13054-022-04041-w (2022).

5 Gardner, A. K. *et al.* The Development of Chronic Critical Illness Determines Physical Function, Quality of Life, and Long-Term Survival Among Early Survivors of Sepsis in Surgical ICUs. *Critical care medicine* **47**, 566-573, doi:10.1097/ccm.0000000000003655 (2019).

6 Griffiths, J. *et al.* An exploration of social and economic outcome and associated health-related quality of life after critical illness in general intensive care unit survivors: a 12-month follow-up study. *Critical care* **17**, R100, doi:10.1186/cc12745 (2013).

7 Thomas, S. & Mehrholz, J. Health-related quality of life, participation, and physical and cognitive function of patients with intensive care unit-acquired muscle weakness 1 year after rehabilitation in Germany: the GymNAST cohort study. *BMJ Open* **8**, e020163, doi:10.1136/bmjopen-2017-020163 (2018).

8 Myhren, H., Ekeberg, Ø. & Stokland, O. Health-related quality of life and return to work after critical illness in general intensive care unit patients: a 1-year follow-up study. *Critical care medicine* **38**, 1554-1561, doi:10.1097/CCM.0b013e3181e2c8b1 (2010).

9 Rattray, J. E., Johnston, M. & Wildsmith, J. A. Predictors of emotional outcomes of intensive care. *Anaesthesia* **60**, 1085-1092, doi:10.1111/j.1365-2044.2005.04336.x (2005).

10 Hatch, R. *et al.* Anxiety, Depression and Post Traumatic Stress Disorder after critical illness: a UK-wide prospective cohort study. *Critical Care* **22**, doi:10.1186/s13054-018-2223-6 (2018).
